# Analog nanophotonic computing going practical: silicon photonic deep learning engines for tiled optical matrix multiplication with dynamic precision

**DOI:** 10.1515/nanoph-2022-0423

**Published:** 2023-01-09

**Authors:** George Giamougiannis, Apostolos Tsakyridis, Miltiadis Moralis-Pegios, Christos Pappas, Manos Kirtas, Nikolaos Passalis, David Lazovsky, Anastasios Tefas, Nikos Pleros

**Affiliations:** Department of Informatics, Center for Interdisciplinary Research & Innovation, Aristotle University of Thessaloniki, Thessaloniki, Greece; Celestial AI, 100 Mathilda Place, Suite 170, Campbell, CA 95008, USA

**Keywords:** analog computing, deep learning, dynamic precision inference, photonic computing, silicon photonics, tiled matrix multiplication

## Abstract

Analog photonic computing comprises a promising candidate for accelerating the linear operations of deep neural networks (DNNs), since it provides ultrahigh bandwidth, low footprint and low power consumption computing capabilities. However, the confined photonic hardware size, along with the limited bit precision of high-speed electro-optical components, impose stringent requirements towards surpassing the performance levels of current digital processors. Herein, we propose and experimentally demonstrate a speed-optimized dynamic precision neural network (NN) inference via tiled matrix multiplication (TMM) on a low-radix silicon photonic processor. We introduce a theoretical model that relates the noise figure of a photonic neuron with the bit precision requirements per neural layer. The inference evaluation of an NN trained for the classification of the IRIS dataset is, then, experimentally performed over a silicon coherent photonic neuron that can support optical TMM up to 50 GHz, allowing, simultaneously, for dynamic-precision calculations. Targeting on a high-accuracy and speed-optimized classification performance, we experimentally applied the model-extracted mixed-precision NN inference scheme via the respective alteration of the operational compute rates per neural layer. This dynamic-precision NN inference revealed a 55% decrease in the execution time of the linear operations compared to a fixed-precision scheme, without degrading its accuracy.

## 1 Introduction

The exponential increase of compute-demanding applications, along with their need for time-of-flight and near-zero energy consumption, has rekindled the analog computing paradigm [1–4] as a way to overcome the digital energy wall. Analog computing pares down the data movement requirements by exploiting the memory cells both as storage and computation elements. Additionally, analog computing engines comprise highly promising approaches for AI processing since they operate with much less power at a higher speed compared to their digital counterparts [5]. The latter becomes even more pronounced when the computing hardware exploits the prodigious primitives of light i.e., ultrahigh bandwidth, low footprint and high energy efficiency, with optical neural networks (ONNs) being at the forefront of research and industrial activities within the last decade [5–7] and promising to accelerate matrix multiplication operations, which form typically the most time- and energy-consuming tasks within inference applications of deep neural networks (DNNs) [8].

Yet, ONNs and analog computing engines in general, come with the price of (i) confined size of hardware implementable computational models [9–18] and (ii) limited bit precision [5, 19], [20], [21]. More specifically, as the complexity of the neural network (NN) models proliferates, so does their size and in turn their total number of required multiply-accumulate (MAC) operations. However, the spatial distribution of the NN parameters encoding devices cannot expand relentlessly, imposing a hardware limitation in the number of encodable parameters the ONN can host. To this end, the latter need to follow the lead traced by today’s TPU and GPU computational models [22, 23], where a limited amount of hardware resources can execute DNNs with significantly higher dimensions. In particular, based on the hardware characteristics, i.e., size, parameters updating speed, and the application requirements, i.e., sample-/batch-wise inference, these computational modes split the matrices into smaller tiles and unroll the complete matrix multiplication operations in the time domain. The tiled matrix multiplication (TMM), performed by means of time division multiplexing (TDM), entails the high-speed update of the matrix element encoding devices, calling for ONNs with high-bandwidth constituent building blocks. Towards this direction, ONNs have to strike the balance between operational speed and scaling, with the majority of the integrated photonic solutions leaning their efforts mainly on the second. On top of the above, the digital-to-ONN computing transition includes the employment of digital-to-analog (DAC) and analog-to-digital (ADC) converters along with the parameters encoding, amplification and processing devices, i.e., modulators, photodiodes (PDs), amplifiers etc., that, inevitably, introduce degradation to the analog accuracy during the inference, since each constituent introduces a relevant noise source that impacts the electro-optic link’s bit resolution properties. The limited bit precision effect can be mitigated during the training process or alternatively, via post-training inference techniques. The former can be accomplished either by incorporating the hardware impairments i.e., noise figures, bit quantization limitations etc., into the training model [24–27], or via the employment of rigid rules in the training phase i.e., low-precision training, binarization of the NN parameters etc. [28, 29]. Yet even though these techniques lead to accuracy improvements, they impose additional complexity and energy trade-offs since the NN need to be retrained in order to be tailored to the employed hardware constraints. On the other hand, in pre-trained networks, analog optical processors can step in effectively when operations can be executed at low bit precision [5, 30]. However, the bit resolution requirements of the NNs are, typically, more rigorous. To this end, post-training techniques i.e., inference averaging, dynamic precision inference etc. [19, 31], [32], [33] need to be employed in order to compensate for the “noisy” analog computations.

In this paper, we demonstrate a speed-optimized dynamic precision NN inference via TMM on a silicon-integrated neuromorphic processor. The 2-input SiPho neuron supports high-rate update of the NN parameters (inputs, weights) encoding, allowing for the effective application of TDM. Towards the speed- and accuracy-inference optimization of a hardware-aware trained NN for the classification of the IRIS dataset, we distinguished and modeled the noise figures of the ONN link and the bit precision requirements of each neural layer. After the model-aware correlation of the required bit precision per layer with the ONN axon bandwidth, we experimentally performed the dynamic-precision NN inference revealing a 55% decrease in the execution time of the linear operations compared to a fixed-precision scheme, without significantly (<1%) degrading its accuracy. Additionally, we validated and quantified the impact of the dynamic-precision post-training inference into the NN accuracy, via the operation of the photonic hardware at different compute rates in the two neural layers. Specifically, we performed the inference of the constituent neural layers via TMM, recording the accuracy of the NN, when the linear operations of its two layers were performed at 2, 16, and 50 Gbaud. As a consequence of the high bit precision tolerance of the 1st neural layer, the software accuracy of 96.6% was obtained during its experimental inference at compute rates up to 50 Gbaud. On the other hand, an accuracy degradation was observed in the noise-sensitive output layer, with the accuracy values of 93.1%, 86.4%, and 68.6% being calculated when it was executed at 2, 16, and 50 Gbaud, respectively, validating the dynamic precision significance within the NN inference.

## 2 Photonic aware techniques towards high speed and high accuracy neural networks inference

The exploitation of light primitives for the high-speed and high-accuracy execution of the space- and time-demanding matrix multiplication operations is, typically, accompanied with multiple requirements on the development of the ONN hardware. In particular, the limitations that are imposed by the analog nature of the data movement and processing within an ONN and the finite number of parameters that a practical silicon photonic chip can host, along with their update rate, predominantly define these requirements. In this regime, the speed- and accuracy-optimization of the NN inference has to proceed along with hardware aware methodologies. In this section, we delve into the inference of NNs whose dimensions exceed the ONN dimensions and present the time division unrolling of its execution via the employment of the TMM technique. Additionally, we study and model the noise sources of an ONN link, correlating the operational rate-dependent total noise figure with hardware’s analog precision. Thereafter, we identify the bit precision requirements among the neural layers and propose a dynamic rate regulation method towards the speed-optimization of the NN inference.

### 2.1 Optical tiled matrix multiplication

**Figure 1:**
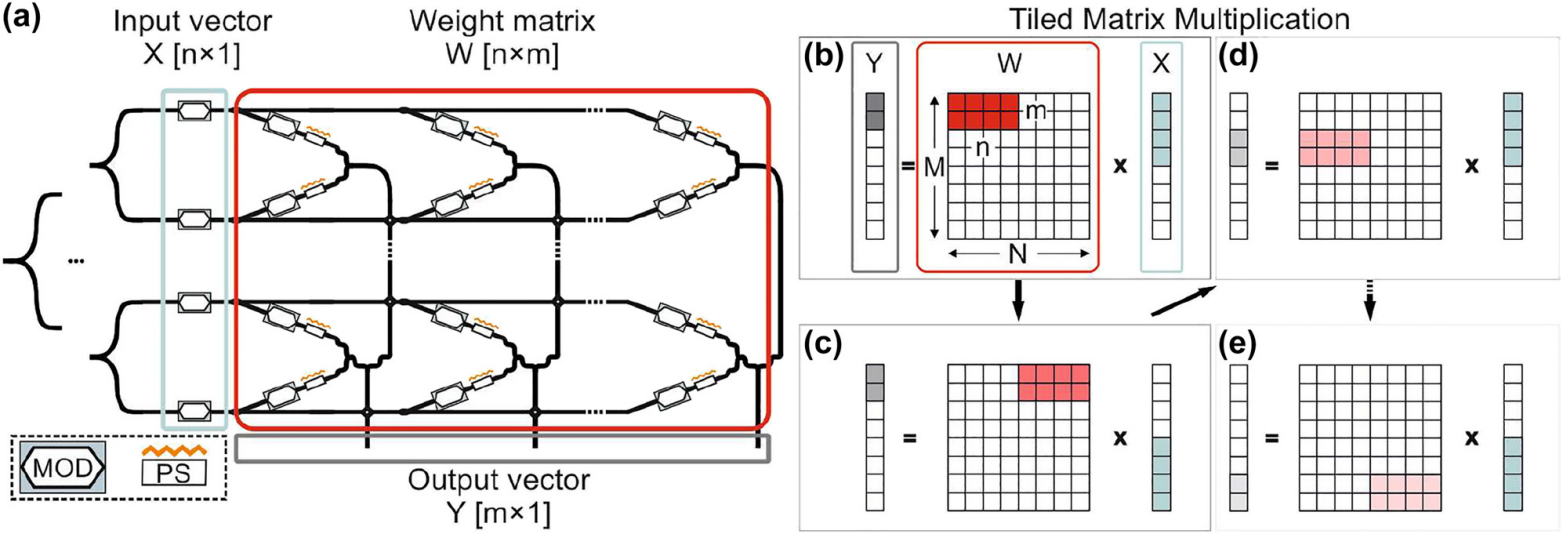
Linear operator architecture and TMM: (a) *n* × *m* crossbar architecture for vector (1 × *n*) matrix (*n* × *m*) multiplication. (b)–(e) Tiled vector (1 × *N*) matrix (*N* × *M*) multiplication via a 1 × *n* vector and *n* × *m* matrix encoding hardware.

The processing speed and accuracy of the NN matrix multiplication linear operations is heavily dependent on the structure, the size and the principles of the employed ONN architecture. Figure 1(a) illustrates a coherent photonic crossbar architecture as proposed in [34]. An n-elements long NN input vector can be encoded via the modulating devices included in the light blue rectangle that follows a 1 × *n* splitting stage. A crossbar mesh, highlighted within the red rectangle, performs the *n* × *m* weight matrix (*W*) elements encoding, via modulators for the amplitude and phase shifters (PSs) for the sign imprinting. Hence, the linear operations between the input vector *X* and the weight matrix *W* produce an m-elements long vector *Y*, shown in the grey rectangle [34, 35]. The architecture of the photonic crossbar of Figure 1(a), offers: (i) direct elements mapping, that leads to easy programmability and optimal representation fidelity among the experimental and the targeted values, as opposed to complex unitary-based architectures [13, 14, 16, 36] where the fidelity is degraded due to their differential path/node losses, (ii) high insertion loss savings, since each light beam travels only through #2 modulating and #1 phase shifting active devices, allowing this way for high dimensions-scaling and the employment of technologies that can provide high-speed elements imprinting, combined with low energy consumption and/or low footprint attributes. However, irrespective of the deployed technologies, the dimension scaling of the ONN architecture is power budget bounded into practical numbers (*n*, *m*), that cannot follow typical NN dimensions (*N* > *n*, *M* > *m*) [37, 38]. As such, wavelength and time division multiplexing techniques have to be enforced either for maximizing the amount of parallel operations or for time unfolding of the operations, respectively. The former has been widely used in the domain of integrated neuromorphic photonics [9, 15, 17, 39] but has still limitations in the number of employable wavelengths and, as such, in the amount of parallelization it can provide. Therefore, TDM comprises the imperative solution for executing linear operations of an NN via ONN hardware with limited dimensions. Figure 1(b)–(e) illustrates the TMM steps required by an ONN of dimensions (*n*, *m*) in order to calculate the linear operations of an NN of size (*N*, *M*). More specifically, the *n* × *m* elements of the weight matrix *W* and the *n* elements of the vector *X*, highlighted with red and light blue color in Figure 1(b), respectively, are imprinted in the ONN’s respective devices at time slot #1. Figure 1(c) and (d) describe the following TMM stages until the final step that is illustrated in Figure 1(e). Evidently, in order for the ONN to perform the TMM operations, the update rate of the modulating devices for the *X* and *W* elements should be synchronized and take place at the highest possible speed in order to provide low-latency calculations and minimize execution time. Therefore, this calls for the development of an ONN architecture that can simultaneously support high-bandwidth active constituents and high scaling credentials [34, 40], since high ONN dimensions minimize the number of tiles of the targeted matrices.

### 2.2 Noise-aware neural network inference speed-optimization

Our recent demonstration of the loss-optimized photonic crossbar architecture (Figure 1(a)) [34, 41] that is capable of retaining high fidelity values even for high insertion node losses has highlighted the feasibility of deploying high-bandwidth photonic components with up to 50 GMAC/s/axon rates in high-radix photonic neuromorphic layouts. In this context, we proceed with the development of an analytical framework that is capable of correlating the available opto-electrical bandwidth of the underlying photonic components with the achieved bit resolution equivalent performance of the ONN, towards: (i) identifying the major physical mechanisms that define the relationship between the achievable rate and the ONN bit precision, (ii) revealing the latency-accuracy trade-offs of high speed ONNs and (iii) concluding to a generic model of mixed-precision NN inference, following the paradigm of electronic NN accelerators [29–32].

**Figure 2:**
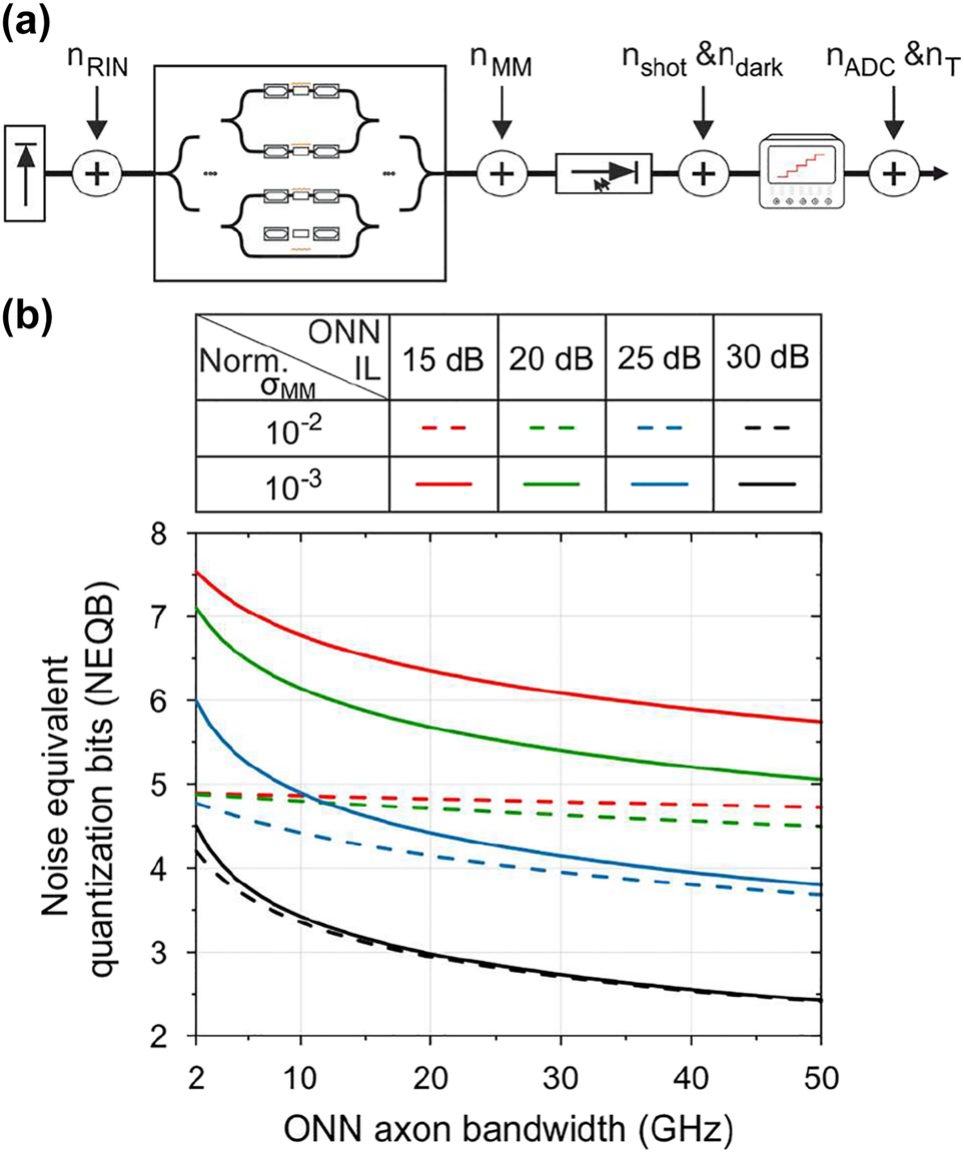
PNN noise analysis: (a) Electro-optic ONN link noise sources breakdown. (b) Noise equivalent quantization bits versus the bandwidth per ONN’s axon for different values of ONN power budget and matrix multiply noise standard deviation values.

We begin our analysis by evaluating the fundamental relationships between the available bandwidth and the achieved bit resolution of an ONN link. Figure 2(a) illustrates a detailed breakdown of the dominant noise sources of a multi-axon neuron link that impact the algebraic product of *X* and *W* via: the *n*_RIN_ that corresponds to the aggregated noise contributions of the laser source, the *n*_MM_ that is related to the matrix multiply electro-photonic link, the shot noise *n*_shot_ that corresponds to the random fluctuation of the PD’s current owning to the discrete charge of electrons traversing the PIN potential barrier, the *n*_dark_ that corresponds to the noise term associated with the finite dark current of a photodetector, the *n*_ADC_ that is correlated with the quantization noise imposed by the limited resolution of the employed ADC components and, finally, the *n*_T_ that is defined as the dominant thermal noise source of the electro-optic layout. Based on the central limit theorem [42], we consider that the dot product calculated via the ONN matrix multiply electro-photonic link follows a normal distribution, introducing a noise term with a standard deviation *σ*_MM_. Additionally, assuming that the shot noise values float above nW levels, the quantization noise is uniformly distributed and the thermal noise is dominated by the input-referred noise of the trans-impedance amplifier (TIA), the aforementioned contributions can be modeled as zero-mean additive Gaussian noise sources and their standard deviations referenced to a photocurrent *I*_avg_ and a noise bandwidth B can be calculated through:

$$\begin{array}{ll} \sigma_{\text{RIN}} & =I_{\text{avg}}\sqrt{\text{RIN}\times B},\\ \sigma_{\text{shot}} & =\sqrt{2\times q\times n_{\text{PD}}\times \left(I_{\text{avg}}+I_{\text{dark}}\right)\times B},\\ \sigma_{\text{ADC}} & =\sqrt{1/12}\times \Delta /\left(2^{\text{Ebits}}-1\right) \end{array}$$

(1) $$\sigma_{\text{T}}=i_{\text{ref}}\times \sqrt{B}$$

where RIN corresponds to the relative intensity noise density, *q* to the electron charge, *n*_PD_ to the conversion efficiency (responsivity) of the PD, Δ to the quantization interval equal to *P*_max_ − *P*_min_, Ebits to the effective number of bits (ENOB) of the employed converter and *i*_ref_ to the TIA input referred noise current density. Additionally, we correlate the *P*_max_ − *P*_min_ values with the modulators’ extinction ratio (ER) through the equation *P*_Avg_ = *P*_max_ − *P*_min_ × (ER + 1)/2 × (ER − 1). Finally, considering the square law detection at the PD and assuming the dark noise’s contribution to be negligible compared to the photodetector’s shot noise, we approximate the standard deviation of the total noise of an ONN link calculated through:

(2) $$\sigma_{\text{TOTAL}}=\sqrt{{\sigma_{\text{RIN}}}^{2}+{\sigma_{\text{shot}}}^{2}+{\sigma_{\text{ADC}}}^{2}+{\sigma_{\text{T}}}^{2}+{\sigma_{\text{MM}}}^{2}}$$

Following Eq. (2), we calculate the noise equivalent quantization bits (NEQB) of the overall ONN link as:

(3) $$\text{NEQB}=\text{lo}{\text{g}}_{2}\left(\Delta^{\prime }/(\sqrt{12}\times \sigma_{\text{TOTAL}\left(\right)}+1\right)$$

adopting the convention that Gaussian noise sources can be correlated to limited bit precision of NNs [19]. In order to quantify the compute rate’s impact on the ONN bit resolution capabilities based on the developed framework, we considered typical values for state-of-the-art high-bandwidth electro-photonic components that include: $\text{RIN}=-150\;\;dB/Hz,\;\;\;\;\;n_{\text{pd}}=0.8\;\;\text{A/W},\;\;\;\;\;\text{ER}=10\;\;dB,\;\;\;\;\;i_{\text{ref}}=1,5E-11\;\;\text{A}/\sqrt{\text{Hz}}$ and Ebits_ADC_ = 8. Given that the received power at an ONN’s output depends on the total insertion loss (IL) of the architecture through IL_architecture_ = *P*_Tx_ − *P*_Rx_, Figure 2(b) illustrates the relationship between the achievable NEQB at an ONN axon bandwidth range of *B* ∈ [2, 50 GHz]. The bandwidth-NEQB correlation was calculated for different typical neuromorphic photonic layout ILs equal to 15 (red), 20 (green), 25 (blue) and 30 dB (black), referenced to a laser emitted power of 16 dBm and considering the normalized standard deviation of the noise of the matrix multiply device equal to *σ*_MM_ = 10^−2^, shown in dashed lines, and *σ*_MM_ = 10^−3^, shown in solid lines. The analysis reveals both the relationship between *σ*_MM_ and the achieved NEQB, as well as the ONN architecture’s IL impact on the achievable bit resolution performance. As expected, the NEQB values follow a decreasing course as the bandwidth and the ONN IL increase. Additionally, one can observe that the impact of the *σ*_MM_ to the NEQB values becomes more intense at lower IL values, while the importance of a loss-saving ONN architecture becomes more evident when the matrix multiplying device’s noise standard deviation remains at low values. On the contrary, as the *σ*_MM_ increases, the impact of the ONN’s IL decreases and the NEQB curves are dominated by the remaining noise sources. More specifically, the thermal noise becomes the limiting noise factor when the total ONN’s IL ranges among high values (>25 dB). As the IL decreases, the RIN originated noise dominates the total noise figure and as such the NEQB.

**Figure 3:**
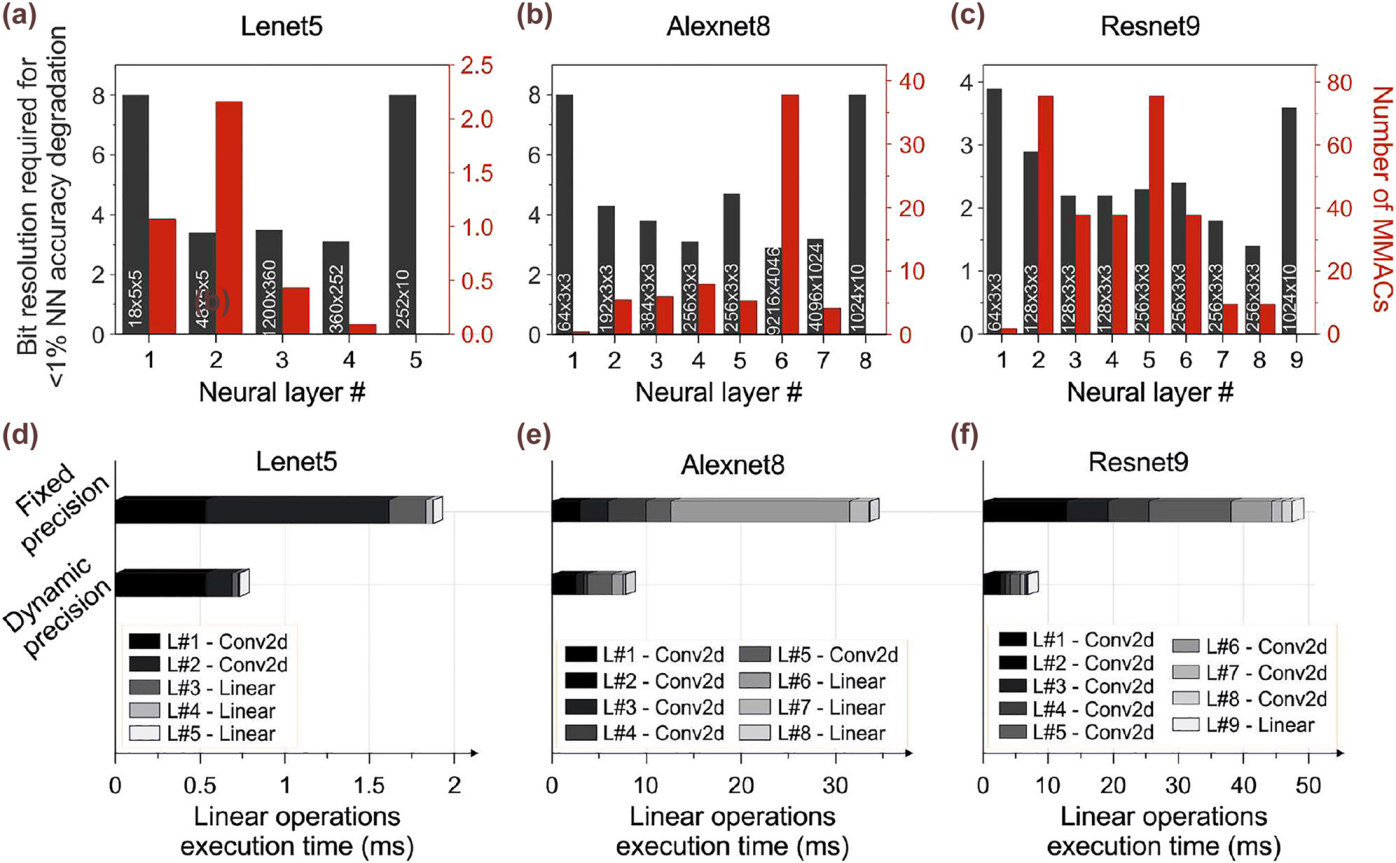
Bit resolution required for <1% NN accuracy degradation (grey bars) and number of MMACs (red bars) per layer of (a) the Lenet5, (b) the Alexnet8 and (c) the Resnet9 NNs. Linear operations execution time in ms for fixed and dynamic bit precision inference at (d) the Lenet5, (e) the Alexnet8, and (f) the Resnet9 NNs.

Towards effectively exploiting the NEQB-ONN bandwidth relation for the latency-optimization of the linear operations of the NN inference, we correlate the individual neural layers’ requirements in bit precision with the overall NN accuracy. More specifically, we examine how accurately the NN performs the inference when each neural layer’s linear operations are, individually, performed with predefined quantization bits ranging within the [1, 8] range. Thereafter, after the identification of the network’s “demands”, we extract the minimum bit precision values that can be tolerated by the NN without significantly degrading the final accuracy and select the compute rate of the linear operations of each layer that, based on our previous analysis, can provide this NEQB. This dynamic-rate NN inference leads to significant execution time savings, which can, eventually, turn into respective energy gains. Towards showcasing the proposed method, we evaluated the bit precision requirements of the individual layers of three pre-constructed popular convolutional NNs, the Lenet5 [43], the Alexnet8 [44] and the Resnet9 [45], that comprise 5, 8, and 9 layers, respectively. The dark grey bars of Figure 3(a)–(c) illustrate the minimum bit precision requirements of each neural layer under the condition that the maximum NN accuracy degradation will not exceed 1% with respect to the maximum achievable value defined by the training process, for the examined NNs, respectively. When the NN accuracy degrades by more than 1%, then we consider that the minimum bits required equal to 8. It can be observed, that each layer performs differently under the bit precision relaxation, with the first and last being the less tolerant layers in all three networks, since information loss in one layer cannot be later recovered in the subsequent ones, according to Data Processing Inequality [27, 46]. In order to quantify the achievable savings in execution time, we, also, extract the number of MAC operations that need to be performed per neural layer, shown in the red bars of Figure 3(a)–(c). Consequently, we calculate and compare the NN inference linear operations’ execution time when the compute rate is fixed to the rate that does not lead to NN accuracy degradation by more than 1% and the dynamic-precision aware NN inference. Figure 3(d)–(f) illustrate the computing times of the neural layers of each of the examined NNs, when the inference follows the fixed- (upper stacked bars) and the dynamic- (lower stacked bars) precision inference methods. The NEQB selection in the latter case was realized based on the metrics that were considered for the calculation of the black dashed line of Figure 2(b) that might approximate high-scale ONN architectures characteristics. Indicatively, the bit precision requirements analysis for the Alexnet8 NN revealed that the execution of the 7.96 MMAC operations of the 4th layer requires at least 3.1 bits of precision, that, based on the developed model, correspond to a minimum compute rate of ∼23.8 GMAC/s, resulting to a total time of execution of ∼0.33 ms. Following the proposed mixed compute rate NN inference, the analysis revealed 61%, 76% and 85% decrease in the aggregate execution times for the Lenet5, the Alexnet8 and the Resnet9 NNs, respectively. These latency-reduction rates may lead to significant energy savings or, eventually, compensate for the latency introduced via TMM techniques, where the ONN’s achievable MAC operations per time slot are inferior to the NN required ones. Finally, it is worth noting that as the number of neural layers increase, the importance of the dynamic-rate NN inference will presumably become more pronounced.

## 3 Dynamic-precision NN inference: experimental setup

**Figure 4:**
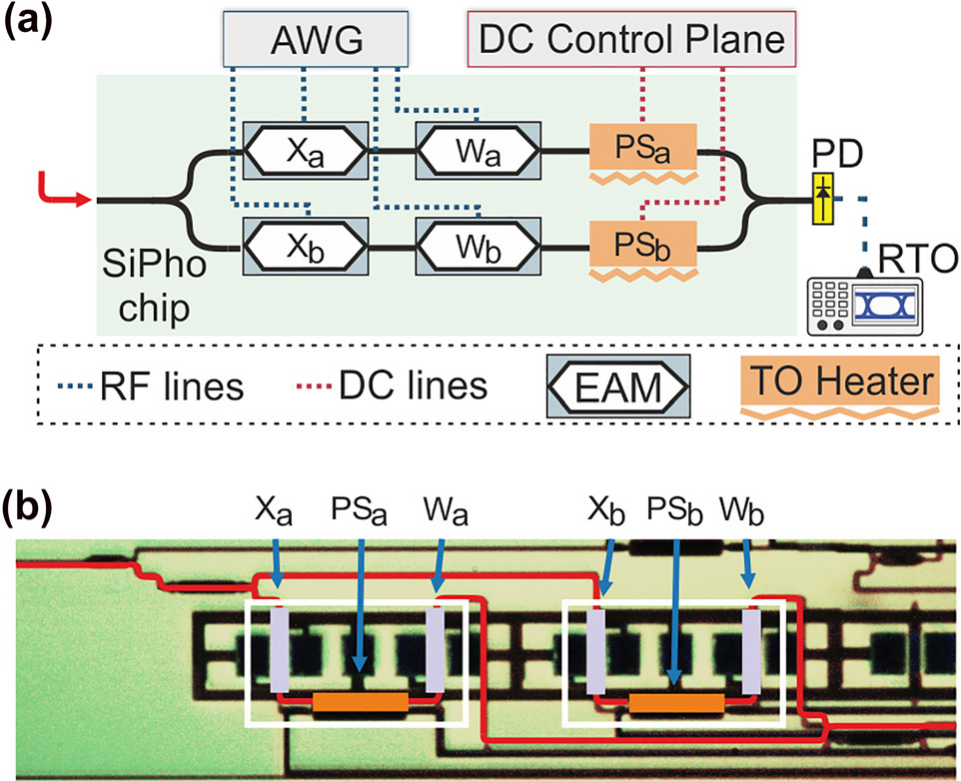
Experimental testbed: (a) Experimental setup established for the inference of the NN for the IRIS dataset classification. (b) SiPho processor employed for the NN inference.

In order to experimentally evaluate the TMM and the dynamic precision in an NN inference, we established the experimental setup shown in Figure 4(a). A light beam at 1560 nm was injected, via a grating coupler with an IL of 3 dB, into the Sipho chip depicted in Figure 4(b), where an electro-absorption modulator (EAM)-based 2:1 single column crossbar processor was designed and fabricated. The optical signal was then split into two identical branches of an MZI, via 3 dB Y-junction multimode interference (MMI) coupler, where, in each branch, two cascaded EAMs, with an IL of 4.4 dBs each, were utilized for transferring to the optical domain the NN input values, while the thermo-optic (TO) PSs were used to statically bias the MZI in the desired operating point. The digital NN inputs were converted in the analog domain via ADCs, using four channels of an arbitrary waveform generator (AWG – Keysight M8194a) and, after amplification, fed to the EAMs in order to be transferred in the optical domain. Specifically, the EAMs, annotated as Xa, Xb in Figure 4(a), were utilized for modulating the input-data of the deployed NN, while the NN weight imprinting was achieved by the EAMs Wa, Wb. Finally, a 3 dB Y-junction MMI was employed for the coherent addition of the two sequences, and then the weighted sum was injected a PD before being captured by to a real time scope (RTO – Keysight DSOZ632a). A digital signal processing stack was utilized both in the transmission and the reception site, including quantization, filtering, resampling, and time recovery. The overall IL of the photonic processor was calculated at ∼15 dB, including the 6 dB losses of the grating coupler based I/O interfaces.

**Figure 5:**
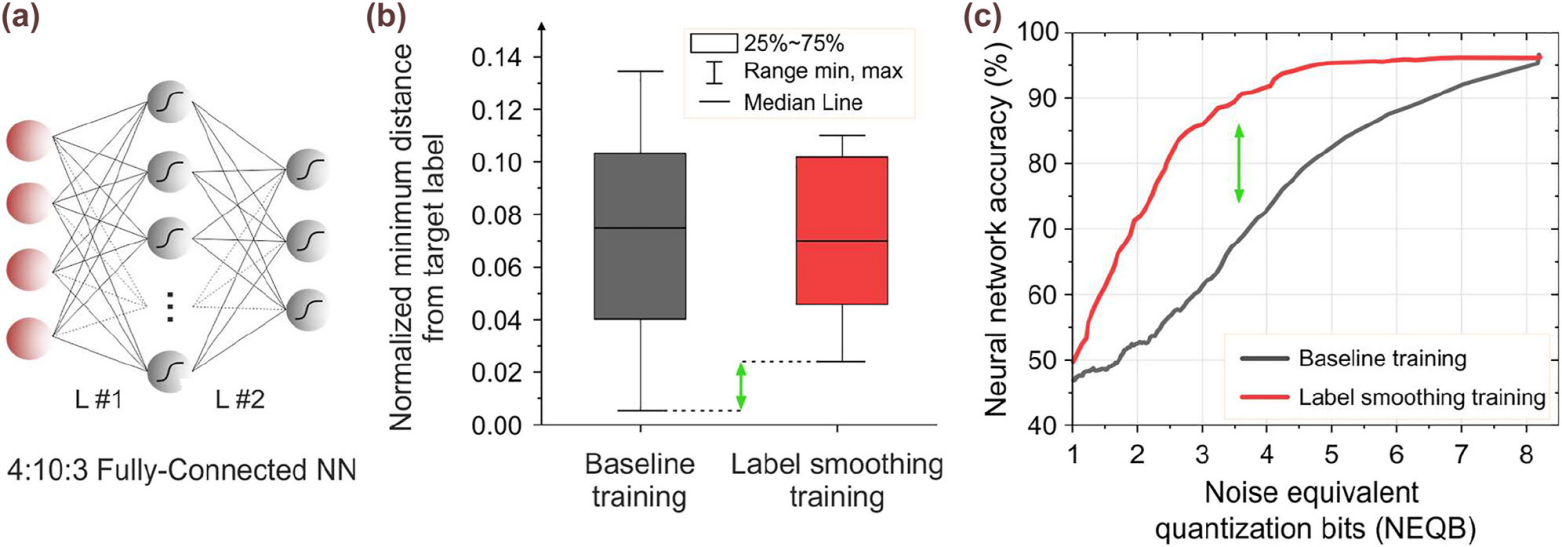
NN training: (a) 4:10:3 fully-connected NN for the classification of the IRIS dataset, (b) Normalized minimum distance from target label *t*_*c*_ and $t_{c}^{mod}\;$ when the baseline and label smoothing training were employed, respectively. The latter increases the minimum distance of the output classes by ∼8 times compared with the baseline training. (c) Classification accuracy versus NEQB when the baseline and the label smoothing training are employed.

In view of benchmarking our Sipho processor into a real DNN application and evaluate the proposed TMM and dynamic-precision inference schemes’ impact, we designed and trained an NN for the classification of the IRIS dataset, whose topology is illustrated in Figure 5(a), comprising a 4:10:3 fully-connected network. Although our architecture has already validated its credentials to support both positive and negative values of the NN input and weighting elements using the deployed PSs to provide the sign information [35], in this work, we enforced all NN parameters to be positive. This mainly stems from the use of TO PSs for the sign imprinting that can’t follow the high data-rate speed (GHz regime) of the input-data and weight imprinting EAMs, as would be required during the TMM operation. This can be certainly overcome either by replacing the TO PSs with available electro–optic PS technology that can support high-speed operation or, in our case, by adopting non-negative NN training models. However, using only positive NN parameters in DL models, poses significant challenges in the training process that have to be addressed in order to yield high classification accuracies. This constraint, typically, generates outputs that the NN struggles to discriminate when baseline training is employed. In order to counteract this effect, we deployed a label smoothing training process [47], turning the output layer more robust to noise and hence to produce more distinctive output classes. In particular, training with label smoothing encourages the activations of the output layer to be close to the template of the correct class and equally distant to the template of the incorrect classes, targeting to minimize the cross entropy, that is defined as $J\left(p,t\right)=-\overset{N}{\underset{c=1}{\sum }}t_{c}\log p_{c}$ , where *N* is the number of classes/output neurons, *t*_*c*_ comprises the true targets and *p*_*c*_ provides the likelihood assigned to the *c*th output neuron. As it can be derived from the equation, the cross entropy is minimized when the likelihood *p*_*c*_ is set to its maximum value. To this end, considering a uniform distribution $u\left(t_{c}\right)=1/N$ , we modified the true targets as: $t_{c}^{mod}=t_{c}\left(1-a\right)+a/N$ , where *a* is used as a hyperparameter. Therefore, employing the label smoothing, the predictions/true targets that refers to the same class form a much tighter cluster, meaning that it eliminates the similarities between the output classes, thus increasing the minimum distance among their values. Finally, due to the non-negativity of the NN parameters, the NN classifier was confined to positive decision boundaries. For this reason, we introduce a linear transformation in the input space of the classifier by utilizing an auxiliary linear layer before the actual network, realizing in this way both positive and negative slope decision boundaries. After adopting the proposed DL training techniques, the NN is optimized for 80 epochs using the AdamW [48] optimizer with a learning rate of 0.01 and a batch size of 32 samples. The classification accuracy that was achieved via the software was 96.6%. Figure 5(b) illustrates the impact of the label smoothing training on the output classes. The *Y* axis depicts the normalized minimum distance from the true target *t*_*c*_ and $t_{c}^{mod}\;$ , when baseline and label smoothing training is deployed, respectively. As it can be observed, using the label smoothing training, the minimum distance among the values of the output classes is increased almost 8 times compared with the baseline training, turning the NN classifier more robust to noise. The latter can also be verified in Figure 5(c), where the proposed training method yields the maximum classification accuracy (96.6%) using ∼5 NEQB, while in order to achieve the same performance in the baseline training the required NEQB was ∼8.

## 4 Dynamic-precision NN inference: experimental results

**Figure 6:**
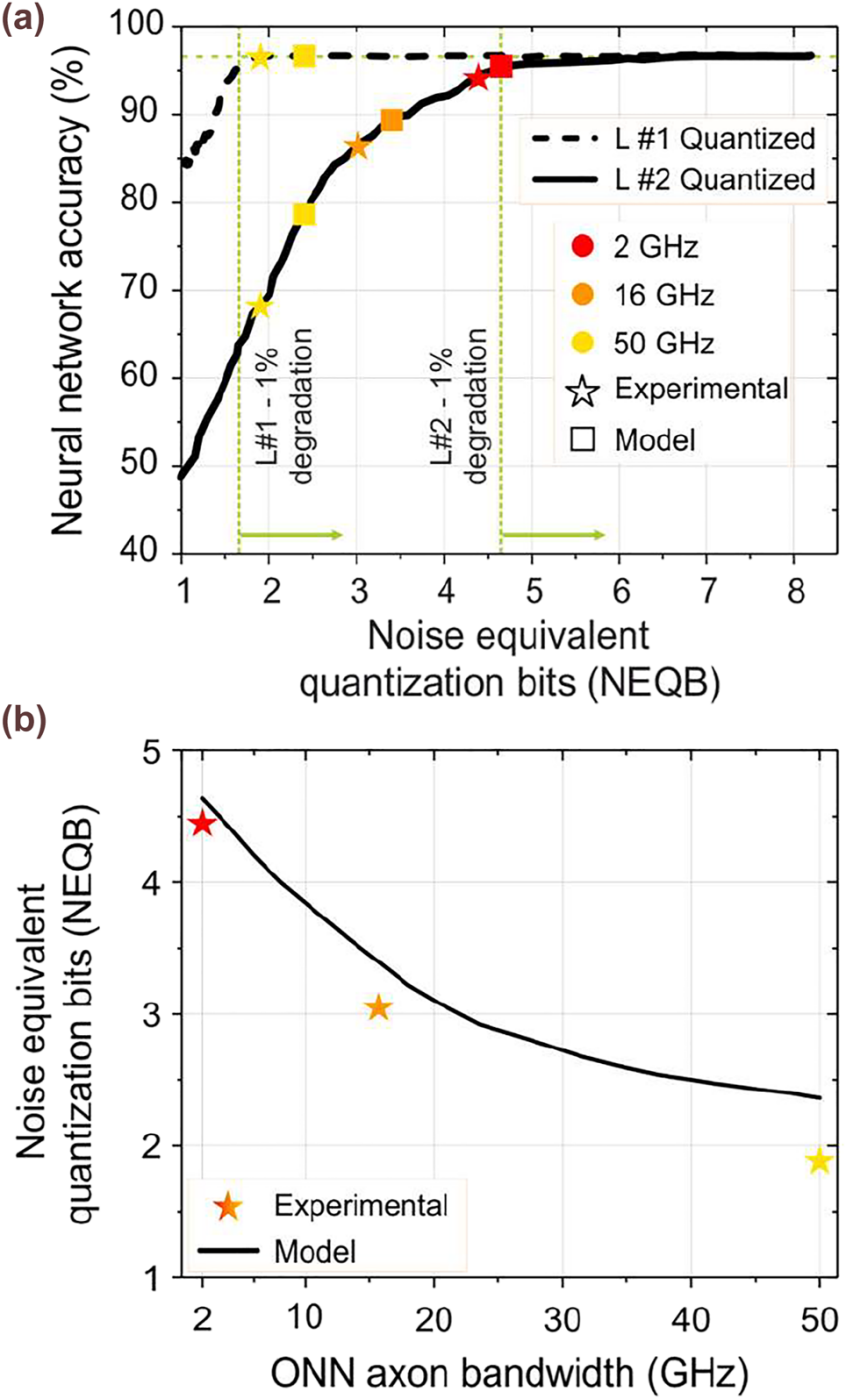
Experimental – model performance comparison: (a) NN inference accuracy when the first (dashed line) and the second (solid line) layers are quantized with a NEQB in [1,8]. Scatter points correspond to the modeled (rectangles) and experimental (stars) respective values at ONN axons’ bandwidth equal 2 (red), 16 (orange), and 50 (yellow) GHz (b) NEQB versus ONN axon’s bandwidth derived via the software model (solid line) and the experiment (star scatter points).

**Figure 7:**
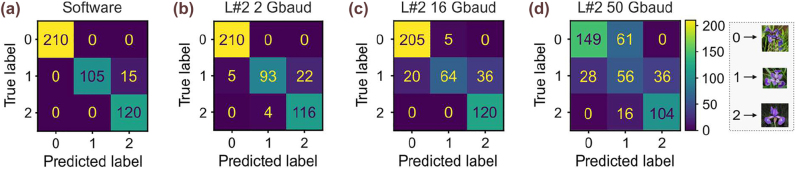
Experimental results: (a)–(d) Confusion matrices derived from the NN inference (a) via software, (b)–(d) when the linear part of the first layer is calculated via the SiPho chip at 50 Gbaud/axon and the second layer at (e) 2, (f) 16, and (g) 50 Gbaud/axon.

In this section we provide our experimental findings on TMM and the adaptable line-rate as they have been obtained during the photonic NN inference of the IRIS classification dataset employing the Sipho processor shown in Figure 4(b). Specifically, the Sipho chip was employed to execute the linear operations of the NN, while the Sigmoid activation function was applied in the software domain. However, in an all-optical implementation the sigmoid activation function could potentially be experimentally deployed using semiconductor optical amplifiers [49]. Within the scope of benchmarking the dynamic precision NN inference, we investigated the NEQB requirements for each neural layer. Specifically, we quantized the NN input and weight parameters of the examined layer in the range [1, 8], with the dashed and solid black lines highlighting the precision requirements of the first and the second neural layer, respectively. Targeting a maximum classification accuracy degradation of up to 1%, i.e., 95.6%, we observed that the 1st layer, being more noise-tolerant, requires low precision calculations of at least 1.6 NEQB to meet the aforementioned condition, as illustrated by the left green dashed line in Figure 6(a). On the other hand, the noise-sensitive output layer requires a NEQB of at least 4.6 to achieve the same performance. Parametrizing the developed theoretical model described in Section 2.2, with the electrical and optical equipment employed in the experiment, we investigated the impact in NEQB as the ONN axon bandwidth gradually increases, as shown with the black curve of Figure 6(b), towards determining the respective compute rate of the constituent neural layers. More specifically, the model parameters included: a RIN = −145 dB/Hz coming from the external laser source utilized (CoBrite-DX Laser type G), a PD with a responsivity of *n*_pd_ = 0.12 A/W with a bandwidth of *B* = 50 GHz, an ER = 10 dB and $\text{AD}{\text{C}}_{\text{Ebits}}=8\;\;\;and\;\;\;i_{\text{ref}}=1,5E-11\text{A}/\sqrt{\text{Hz}}$ from the employed RTO. As observed, the RTO’s noise floor comprised the dominant source of the noise figure of the ONN link. The NEQB-ONN bandwidth correlation in combination with the NEQB-NN accuracy analysis comprised the key for the effective selection of the inference compute rate per layer, towards the post-training NN inference speed- and accuracy-optimization. Targeting at a NEQB ⩾ 1.6 bit, as extracted from the model, the ONN can operate at the maximum available bandwidth of 50 GHz for the computation of the 1st layer. On the other hand, due to its increased NEQB requirements and the model derived metrics, the linear operations of the 2nd layer need to be performed at 2 Gbaud in order for the classification accuracy to be retained at high values. During the experimental dynamic-rate NN inference, our Sipho processor yields indeed the maximum classification accuracy of 96.6% when having its 1st layer operating at 50 Gbaud, suggesting that a NEQB > 1.6 was achieved even in the experimental domain. At the same time, an accuracy degradation of 3.3% was observed when the linear operations of the 2nd layer are experimentally executed at 2 Gbaud. This deviation from the theoretically predicted value becomes even more pronounced as the compute rate increases, with an 86.4% experimental accuracy observed at 16 Gbaud instead of the theoretically expected 89% and a 68.6% experimental accuracy instead of 79% for the 50 Gbaud operational regime. These deviations probably originate by the assumption for the exclusive presence of non-deterministic noise sources in our model, with all noise sources simulated as Additive White Gaussian Noise (AWGN). Yet, as the compute rate increases and approaches the available bandwidth of the deployed Sipho processor, the contribution of the deterministic noise sources is enhanced, as this mainly owes to the limited bandwidth of the photonic and the electronic components. The classification accuracy-NEQB-ONN bandwidth divergence between the theoretical projections and the experimental performance can also be clearly illustrated in Figure 6(a) and (b), respectively, via the red, orange, and yellow star and rectangle scatters that correspond to the 2, 16, and 50 GHz values, respectively. The dynamic-rate NN inference has also significant benefits in its overall execution time. Given that 40 and 30 MAC operations need to be implemented in the 1st and 2nd layer of the IRIS dataset, respectively, we can conclude that the execution time is decreased by ∼55% when the dynamic-rate NN inference is employed over the conventional fixed-rate NN inference, when the latter is performed in the maximum compute rate that is capable of achieving the same accuracy target values as the dynamic-precision scheme, i.e., 2 Gbaud. Finally, Figure 7 provides a pictorial representation of the samples classification via the confusion matrices derived from the execution of the IRIS dataset. Initially, the total samples of this dataset were 30, which comprises a rather poor statistical interpretation towards benchmarking our photonic processor. For this reason, we reused the original samples 15 times during the inference process and we calculated the classification accuracy considering 450 samples in total. Figure 7(a) depicts the confusion matrix acquired from the software, where only 15 out of 450 instances were incorrectly classified that corresponds to a classification accuracy of 96.6%. Figure 7(b), (c) and (d) illustrate the experimentally derived confusion matrices at 2, 16 and 50 Gbaud, respectively. As expected, as the compute rate increases so does the false instances, leading in this way to accuracy degradation, with 2 Gbaud yielding 93.1%, 16 Gbaud 86.4%, and 50 Gbaud 68.6%.

## 5 Conclusions

We demonstrated an analog silicon photonic engine and its capabilities to perform TMM and dynamic precision inference among neural layers towards latency-optimized DL accelerators. Initially, we proposed an optical TMM method using TDM with the ultimate target being the execution of high dimension NNs via low-radix ONN hardware. Following, a detailed theoretical model was developed, associating the noise sources and the bandwidth of an end-to-end ONN link with the noise equivalent bits. In the scope of speed- and accuracy-optimizing the inference of NN linear operations, we trained an NN for the classification of the IRIS dataset and experimentally applied a dynamic-precision inference via an integrated SiPho ONN using TMM. After breaking down each neural layer’s bit precision impact on the overall classification accuracy, we extracted the NEQB requirements in order for the NN accuracy not to be degraded by more than 1% and correlated with the ONN’s bandwidth via the developed model. Thereafter, following a dynamic-rate inference we experimentally computed the 1st neural layer at 50 GHz without imposing any degradation at the software acquired classification accuracy of 96.6%. With the output layer being more sensitive to the noise the experiment revealed a compute rate-dependent accuracy that was calculated equal to 68.6%, 86.4% and 93.1% when its linear operations were computed at 50, 16 and 2 Gbaud/axon, respectively, closely matching the modeled-expected values. Finally, we derive that the execution time benefits by the employment of the dynamic-precision NN inference, for the classification of the IRIS dataset, approximate to 55% compared to a fixed-precision scheme, without introducing any degradation to its accuracy.
